# From construction machines to remote construction robots: control, interfaces, and usability of the Cranebot 

**DOI:** 10.3389/frobt.2024.1504317

**Published:** 2025-01-03

**Authors:** Alessandra Duz, Francesca Negrello, Alexandr Rucodainii, Daniel Lanzoni, Mario Corsanici, Angelo Iapichino, Andrea Vitali, Daniele Regazzoni, Valentino Birolini, Roberto Signori, Rossano Ceresoli, Giorgio Grioli, Antonio Bicchi, Manuel G. Catalano

**Affiliations:** ^1^ SoftBots, Istituto Italiano Di Tecnologia, Genova, Italy; ^2^ Fassi Innovation Center, Nembro, Italy; ^3^ Dipartimento Di Ingegneria Gestionale, Dell’Informazione E Della Produzione, Università Degli Studi Di Bergamo, Dalmine, Italy; ^4^ Consorzio Intellimech, Bergamo, Italy; ^5^ Centro Di Ricerca E. Piaggio E Dipartimento Di Ingegneria Dell’Informazione, Università Di Pisa, Pisa, Italy

**Keywords:** construction robotics, remote control, intuitive interfaces, usability, hydraulic systems

## Abstract

Construction machines, for example cranes, excavators, or bulldozers, are widely diffused systems operating outdoors in harsh and dangerous environments, such as building sites, forests, and mines. Typically, construction machines require the on-site presence of highly skilled users to manage the complexity of their control and the high power of hydraulic actuation. Construction machines could benefit from recent developments of robot avatar technology that has demonstrated the viability of remotizing human physical activities, leveraging on intuitive interfaces and controls. Similar approaches could also improve the overall usability of construction machines, making them safer and accessible for untrained users. With this in mind, we developed a novel system for the remote control of cranes through intuitive and immersive interfaces. To validate the solution, we evaluated the experience of approximately 80 untrained users that remotely operated a crane during the 33rd Edition of Bauma, the world’s leading fair for construction machines.

## 1 Introduction

Construction machines are heavy duty systems that perform a large variety of tasks, like digging, cutting, demolition, lifting, or earth moving, depending on their configuration. Their movements are commonly generated by a series of hydraulic actuators that the user has to control joint-by-joint to perform the desired motion of the terminal part, which is usually referred to as the boom tip. This results in a non-linear system complex to be managed, which requires extensive operator experience on the specific machine model to fully exploit their potential. Moreover, operators are typically working outdoor, often in proximity with other construction machines, which requires high concentration levels for both perceiving and understanding the surrounding environment and governing the machine’s movements. System complexity and hard working conditions are leading to a general workforce shortage in developed countries (Brucker Juricic et al., 2021).

These reasons are a strong push for the manufacturers of construction machines, and specifically cranes, toward the introduction of automation in their systems to ease their use; for example, developing Cartesian control of cranes’ tip (known in the construction field as the crane’s boom tip control) to autonomous coordinate of the crane’s actuation. First examples of boom tip control date back to 2013 with John Deere Forestry Intelligent Boom Control (ICB) for forestry applications (Sokolov et al., 2023), and then, in 2018, we had “PONSSE Active Crane” (Ponsse news, 2022) and “Smart Control” (Palfinger smart control, 2022). Multiple studies, such as Manner et al. (2019) and Sokolov et al. (2023), show that the introduction of boom tip control reduces the operation times of the crane. Similar developments could also be found in other hydraulic machines such as excavators (Hutter et al., 2017; Egli et al., 2022).

The above-mentioned applications require reliable control of the piston movement in the face of complex non-linear systems, with high variability due to the mounting of the machine’s components, especially hydraulic valves, and the level of wear. Possible choices to control the hydraulic actuators include linear controllers (Zhidchenko et al., 2023), ultra-local models La Hera et al. (2023), non-linear optimal control (Rigatos et al., 2020), sliding mode controllers Pencelli et al. (2019), learning by demonstration methods (Lee and Brell-Cokcan, 2020), or reinforcement learning techniques (Egli et al., 2022; Jud et al., 2019). Improvements in tracking performance come at the cost of higher computational load, more demanding identification procedures, and additional sensorization.

Another challenge of the construction industry concerns operator’s safety. To this aim, Caterpillar is developing the Cat Command remote station (Rubenstone, 2021), which connects a machine on site with an exact replica of the control cabin over internet. A similar work of ETH (Hutter et al., 2016) enhanced the cabin replica with a simulation of the machine inclination.

The efficacy of this solution could receive a significant boost in terms of immersivity and intuitiveness from recent advances of robot avatar technology (Lentini et al., 2019; Negrello et al., 2017), as demonstrated during the ANA Avatar XPRIZE competition (Behnke et al., 2023).

The goal of our work is to investigate the feasibility of transforming construction machines into remotely controlled systems, improving their overall usability by means of intuitive interfaces and Cartesian control. To explore this approach, we focus on articulated cranes, which are a type of construction machine dedicated to lifting heavy loads and are deployed in different fields, ranging from construction sites to logistic, railway, forestry, mining, recycling industries, and road clearance, just to mention a few. Differently from tower and telescopic cranes, they are characterized by a fixed hook and a several degrees of freedom, which give them the versatility to perform various tasks, especially in constrained environments (Centurycranes, 2023). At the state of the art, there are still fewer studies regarding their automation and remotization as they are more complex compared to other kinds of crane-lift machines, such as tower cranes (Zhu et al., 2023).

This work regards the remotization of the crane control to enhance safety and working conditions of the crane’s operators. We target to address some open points in the literature that concern the design of the teleoperation station by means of introducing immersive human–machine interfaces and the evaluation of their impact on user experience involving crane operators (Chi et al., 2012; Yu et al., 2021; Top et al., 2020). This is achieved by joining the development of boom tip control and the usage of immersive and first-person experience interfaces as enablers for the operators to control the crane movement remotely in an intuitive manner, including the possibility of having different perspectives on the scene (global and detailed point of view).

In the paper, we present the proposed Cranebot architecture and control requirements, such as low computational structure, high adaptability to model uncertainties, and a limited tracking error. Our solution was successfully tested on two Fassi crane models, M20 and F1150, which largely differ in terms of DOFs, payload, and size. Extensive tests have been conducted during the 33rd edition of Bauma World Construction Fair, operating an M20 crane located in Bergamo from our stand in Munich (approximately 350 km between the crane and operator) over internet connection. During this event, we involved crane operators and various professionals in the construction field in the usability test of our system.

This work was developed within the JOiiNT LAB^1^, a joint laboratory established between IIT and Consorzio Intellimech, which is dedicated to applied research and technological transfer. The company Fassi Gru S.p.A. collaborated on the definition of the case study and the technical development.

## 2 Remote Cranebots

This work focuses on two important aspects to improve the crane’s usability:

• Control intuitiveness: this means simplifying the way the user controls machine movements and perceives the environment to reduce the cognitive effort needed to operate the system and widen usability for non-specialized users. This simplification should pair or even improve the crane’s task performance.
• Operator remotization: operating the machine from a distance creates multiple possibilities, such as keeping the operator in a safe/comfortable environment and eliminating the travel time of the operator between different construction sites.

**FIGURE 1:**
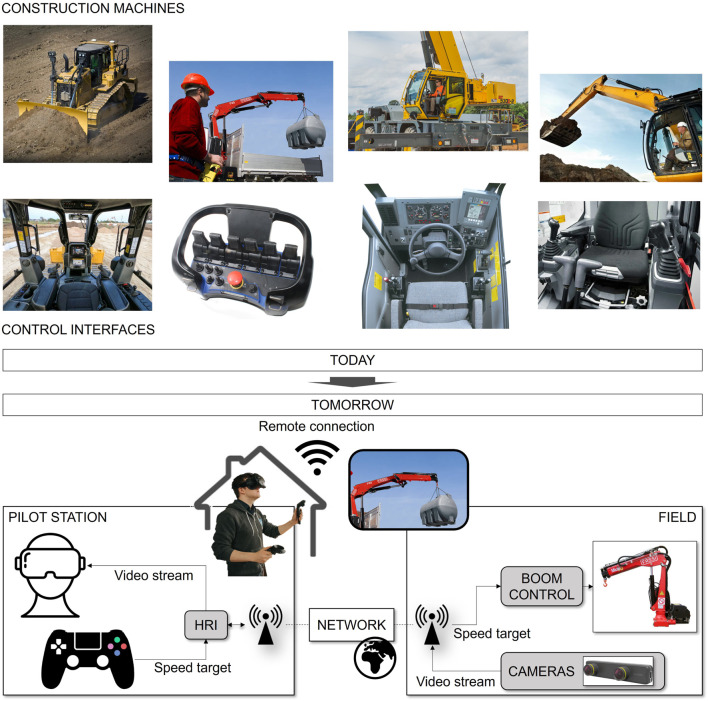
Figure showing the general concept of the investigated solution; we intend to move from on-site joint by joint machine control to off-site intuitive machine operations.

We intend to obtain those two goals through a novel system architecture, as shown in Figure 1. Nowadays, the crane’s operator works in the proximity of the crane with a single point of view corresponding to the operator’s eyesight and a control action on the single actuation of the crane. For the future, we envision an operator being remotely connected with the crane, with the possibility of receiving visual feedback from multiple cameras surrounding the task and directly controlling the movement of the crane boom tip. The constitutive modules of this architecture are the following:

• Crane boom tip control: autonomous coordination of the machine actuation to track a speed reference for the boom tip. This reduces the effort and training required to coordinate the actuators’ movements to obtain the desired boom tip trajectory.
• Human–robot interfaces (HRI): a VR headset will provide a transparent connection to the crane for remote operation, thanks to wearability and immersivity; moreover, its joysticks provide intuitive control interfaces.

Another enabler of remotization is a stable network infrastructure. The presence of delays and packet loss in the exchange of information between the crane and operator has a significant negative impact on the performance; therefore, a suitable network infrastructure should be chosen to limit those effects. Although we faced this technological challenge and we report our experience, an extensive discussion is out of the scope of this paper.

The following sections will deepen the constitutive modules of the system, the technological choices, and usability test of the proposed solution.

### 2.1 Cranebots’ architecture

Our control module autonomously regulates the pistons’ movement in response to a boom tip speed command so that the operator can focus uniquely on the crane’s global task. Here, we discuss all the aspects related to the development and implementation of the boom tip control on standard cranes. Please note that we do not consider the load hanging movements since it is a widely discussed topic, and various solutions already exist to plan the boom tip path to minimize the load oscillations (Ramli et al., 2017).

#### 2.1.1 System model

**FIGURE 2:**
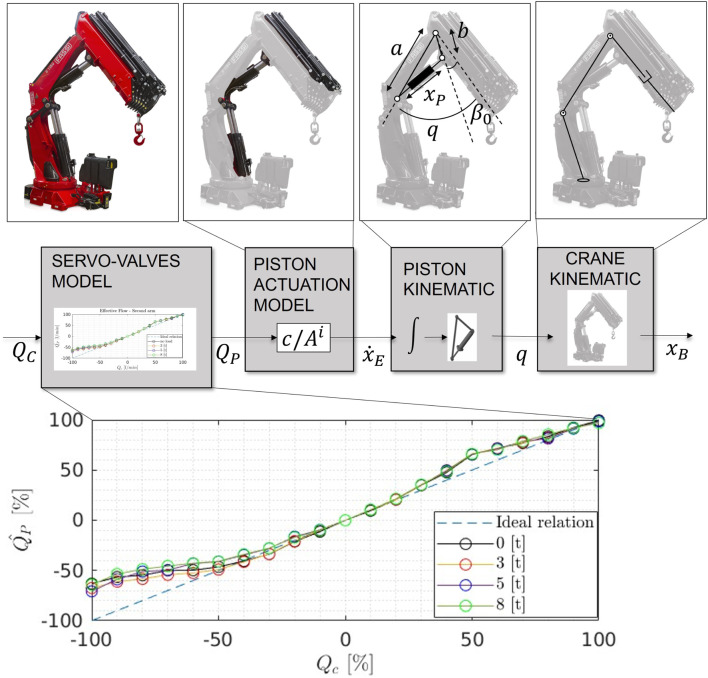
Scheme of the crane system model: from the pump request $Q_{c}$ to the boom tip movement $x_{B}$ .

The boom tip control relays on a crane system model (Figure 2) describes the relation between the actuation flow and boom tip movement, and its main components are explained herein.

Crane kinematics: under the assumption of negligible deformation, the crane structure can be featured as a series of rigid bodies connected by linear or rotational joints that can be modeled with standard conventions, such as Denavit Hartenberg parameters. With the knowledge of the crane kinematics homogeneous transformation matrix, we can easily relate any configuration of the joints’ position $\bar{q}$ with the position of the boom tip of the crane $x_{B}$ .

Piston kinematics: the relation between the pistons’ actuation $\left(x_{P}^{i}\right)$ and the crane’s joints $\left(q^{i}\right)$ is a non-linear analytical function that depends on the specific mechanism implemented. As an example, Figure 2 shows a possible structure for the piston kinematics (Equation 1). From simple geometric considerations, we can derive the relation between the angle q and the piston elongation $x_{P}$ as follows:

$$q=\beta_{0}+\arccos\left(\frac{a^{2}+b^{2}-x_{P}^{2}}{2ab}\right),$$ (1)

where $\beta_{0}$ represents the fixed mechanical offsets between the kinematic model of the piston actuation and the kinematic model of the crane.

Piston actuation: it can be modeled as a spring-damper system, whose equilibrium point $x_{E}^{i}$ is regulated by the flow coming from the pump. Neglecting the fluid compressibility, the speed of the fluid inside the piston is homogeneous as the piston chambers have a cylindrical shape with constant section areas. As a result, the speed of the piston’s equilibrium ${\dot{x}}_{E}^{i}\;\left[m/s\right]$ matches the one of the fluid and can be linearly related to the flow of the fluid entering the piston $Q_{P}^{i}\;\left[\%\right]$ , which is expressed as a percentage of the maximum pump flow, as follows:

$$\begin{array}{c} \;{\dot{x}}_{E}^{i}\;=\;\frac{cQ_{P}^{i}}{A^{i}}\\ c\;=\;\frac{Q_{\mathrm{MAX}}}{100}\frac{1}{60000}, \end{array}$$ (2)

where $A^{i}$ is the piston annulus area $\left[m^{2}\right]$ of the piston section with incoming fluid, $Q_{\mathrm{MAX}}$ is the maximum flow that can be delivered by the pump, and c is the conversion factor. The overall piston movement can be modeled as $x_{P}^{i}=x_{E}^{i}+x_{O}^{i}$ , where $x_{O}^{i}$ are the oscillations generated by the damp-spring behavior of the piston. We choose to neglect those oscillation components in the piston model and focus only on the equilibrium level of the piston so that $x_{P}^{i}\approx x_{E}^{i}$ .

Hydraulic servo-valves: the hydraulic servo-valves regulate the circulation of the oil flow from the crane’s pump to the pistons, and they respond to a request of flow percentage from the control unit. The control module can regulate the activation command with a resolution of 1% of the total valve opening variation. Although the control command determines a specific amount of fluid passing through, some small non-linearity exists due to fluid losses, variations in the mounting of the valves, and dead zones.

We model this non-linearity with a static function. For its identification, we collected the speed response of each piston for a set of flow requests and for different loads, evaluating an average value after the exhaustion of the acceleration transient. Then, we derived from Equation 2 the estimate of the actual flow and mapped it to the request. The identified relation is independent from the load for all joints. This implies that the effects of fluid compressibility are negligible over the load variation and that the distributor is capable of delivering the same amount of fluid for different power requests. Figure 2 shows an example of the identified servo-valve model for the secondary piston of a model F1150 Fassi crane for load weights ranging from 0[t] to 8[t] over a maximum capacity load of 22[t] with the crane’s extensions completely retracted.

#### 2.1.2 System setup

For enabling a boom tip control solution over a conventional crane, it is required to equip it with few supplementary components:

Sensors: it is necessary to have a direct or indirect measure of all the linear and angular displacement of the crane joints. We choose IMUs for the angular measures of the inclination of the crane’s links (accuracy ±0.3° ), wire-actuated encoders for linear displacement of the crane’s extensions (accuracy ±0.1[mm] ), and rotational encoders for the base rotation (repeatability ±0.6%FS ). The sensors’ measures are collected by the embedded control unit.

Computational unit: the crane boom tip control requires a computational capacity that may exceed the performance of the embedded control unit currently in use. A possible solution is to add an external computational unit, ensuring appropriate frequency communication ( 20[Hz] for our setup) with the embedded control unit of the crane to receive the crane’s state measures and set the opening level command of the servo-valves that regulate the piston’s flow. We used a personal computer with Intel core i7, CPU 1.8[GHz] , and RAM 15[GB] , with operative system Ubuntu 20.04. Note that our choice is not representative of the minimal required computational performance as we did not stress it.

### 2.2 Cranebot control

**FIGURE 3:**
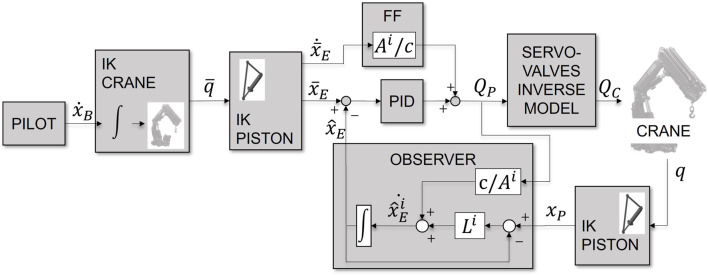
Scheme of the boom tip control structure.

The control module tracks a target speed command for the crane boom tip through the regulation of the servo-valve control action (Figure 3). The speed command is set directly by the crane operator. In Section 2.3, we will provide a deeper focus on the physical interfaces for its selection.

Crane inverse kinematics: the crane boom tip control first integrates the Cartesian boom tip velocity target within the given workspace limits and turns it into a reference Cartesian pose. The availability of an open chain kinematic model allows the application of well-known CLIK techniques, which map the Cartesian target into an array of reference joints’ positions. As the loads attached to the hook are self-orienting in most cases, we adapt the classic inverse kinematics to track only the boom tip position and not its orientation.

Piston inverse kinematics: a dedicated module solves the non-linear kinematic equations that relate the joints’ targets $\left(\bar{q}\right)$ to pistons’ elongation references ${\bar{x}}_{E}$ . In this way, we can directly exploit the linear relation described in Equation 2 for the control action.

Piston equilibrium tracking: we choose to track the piston equilibrium, which determines the position of the boom tip at convergence, and we exclude the high frequency oscillation from the control due to the fluid elasticity. This choice simplifies the control structure, which is also computationally light, and requires limited sensorization of the system and little identification procedures despite the presence of significant model uncertainties, a highly non-linear system, and actuation delays. Clearly, this comes at the expense of the tracking performances since we are neglecting the transient behavior of the crane; the acceptability of the performances degradation will be discussed in the validation Section 2.2.2.

The control input is a piston flow request that includes the sum of a feed-forward contribution and a close-loop contribution. The former refers directly to the pistons’ model, which is reported in Equation 2. Although this contribution plays an important role in improving the tracking performance, it is not sufficient since the model does not perfectly describe the system, and consequently, we incur integration errors. The close-loop contribution compensates for it by using an estimate of the piston equilibrium. For the estimate of the piston equilibrium, we applied a Luenberger observer on the piston measure, which filters both the crane’s elastic oscillations and the sensors’ noise.

Servo-valve inverse model: the last block compensates for the servo-valves non-linearity by introducing a look-up table based on the inversion of the identified relation between the flow request and actual piston’s flow.

#### 2.2.1 Control stability

In the proposed control structure, the presence of the observer and the servo-valve inverse model compensate for the non-linearities due to the fluid compressibility and the servo-valve system, respectively. Thus, the close-loop action is limited to the linear relation between the fluid entering the piston and the equilibrium of the piston elongation, which can be modeled as an integrator with gain $\frac{c}{A^{i}}$ . The tuning of the regulator has to take into account the presence of significant actuation delay due to the low frequency communication of the embedded control unit and the actuation inertia. To estimate this delay, we observed the time difference between the transmission of the actuation command and the detection of a change in the piston measure for each joint. The result is approximately homogeneous among joints, while it is quite different between crane models as the pump capacity and crane mechanical and hydraulic inertia can change significantly. We choose to limit the PID structure to the proportional contribution only; given the estimate of the delay τ and a choice of safety limit for the gain margin $\bar{\phi_{m}}=60{}^{\circ}$ , the proportional gain for the $i^{th}$ joint is limited by $P^{i}\leq \frac{90-\bar{\phi_{m}}}{180}\pi \frac{A^{i}}{c}$ .

#### 2.2.2 Control validation

To assess the scalability of the proposed control, we tested it on different Fassi crane models characterized by different sizes, DOFs, and payloads. The M20 model has three DOFs, 4[m] of maximum elongation, and 20[kNm] of maximum torque. The F1150 model has four DOFs, 19.70[m] of maximum elongation, and 1000[kNm] of maximum torque. Note that, for porting the code from one machine to another, it is necessary to input the control system its kinematics and related parameters and the servo-valve empirical model resulting from identification.

We observed an actuation delay of 0.2[s] for the M20 model and 1[s] for the F1150 model. For both cranes, we evaluated the Cartesian pose tracking performance of the Cranebot control for a sequence of sinusoidal speed profiles, which were given as input sequentially to each Cartesian coordinate, with total target displacement for each Cartesian direction of approximately 0.3[m] and with the maximum speed module of 0.1[m/s] for the M20 model and 0.05[m/s] for the F1150 model. Note that, although we move the boom tip along one coordinate at the time, the control is actively coordinating and controlling all the crane’s actuations to track the overall position target. Experiments start from a Cartesian position that we consider realistic for a generic pick-and-place activity. All tests are performed without load attached.

**FIGURE 4:**
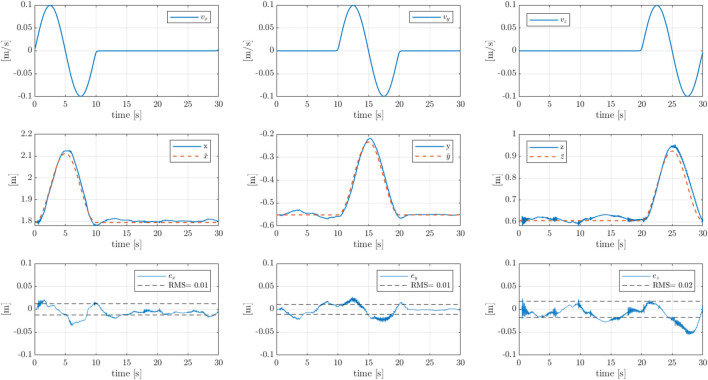
Pose tracking results for the Cartesian boom tip control on the M20 model, where the x,y,z directions are aligned with to the crane’s base frame, as defined in Figure 8. From the top to the bottom are the sequential inputs of Cartesian speed targets along the three Cartesian directions, the Cartesian pose tracking, where $\left[\bar{x},\;\bar{y},\;\bar{z}\right]$ are the boom tip target position, and [x,y,z] the measured positions, and the Cartesian tracking error.

**FIGURE 5:**
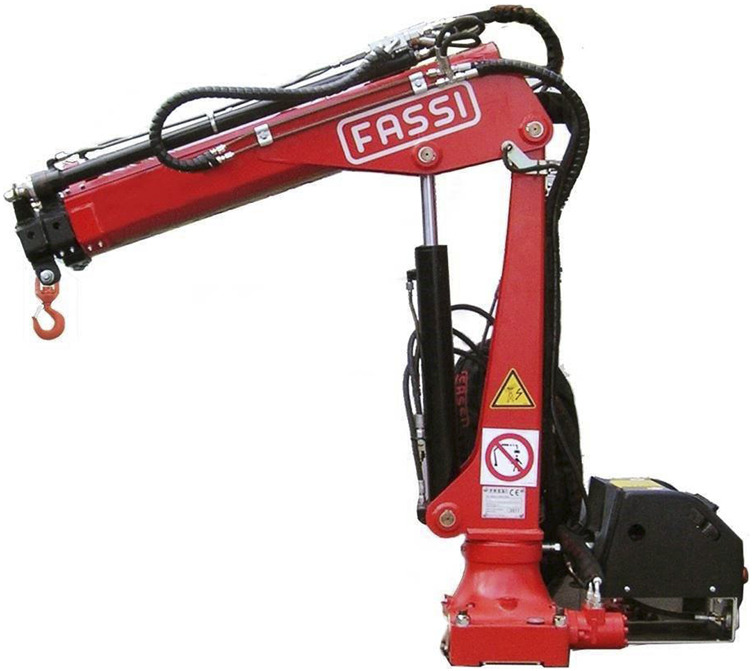
M20 model: 3 Dof reachability: 4 [m] Max. torque: 20 [kNm].

Figure 4 presents the tracking performance for the M20 model, which is shown in Figure 5. From the top to the bottom are the speed input reference, the comparison between the target pose resulting from the speed integration and the actual pose of the crane, and the tracking error. We observe a RMS of approximately 0.01[m] for the x and y directions, with peaks of 0.03[m] in correspondence with the change of direction. This is a direct consequence of the mechanical and hydraulic inertia of the crane. We draw similar consideration for the z direction, and in this case, the overall RMS is equal to 0.02[m] . We observe a degradation of tracking performance in correspondence to a negative speed along the z direction, with a peak of 0.05 tracking error. This effect is due to the presence of a dead zone at the second joint that prevents the fine-tuning of the control action.

**FIGURE 6:**
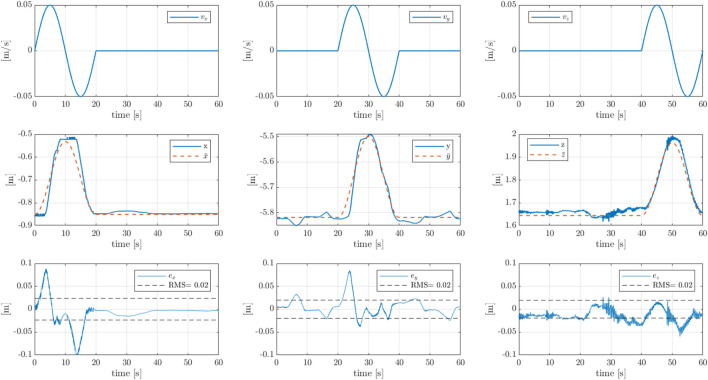
Pose tracking results for the Cartesian boom tip control on the F1150 model, where the x,y,z directions are aligned with to the crane’s base frame, as defined in Figure 8. From the top to the bottom are the sequential inputs of Cartesian speed targets along the three Cartesian directions, the Cartesian pose tracking, where $\left[\bar{x},\;\bar{y},\;\bar{z}\right]$ are the boom tip target position, and [x,y,z] the measured positions, and the Cartesian tracking error.

**FIGURE 7:**
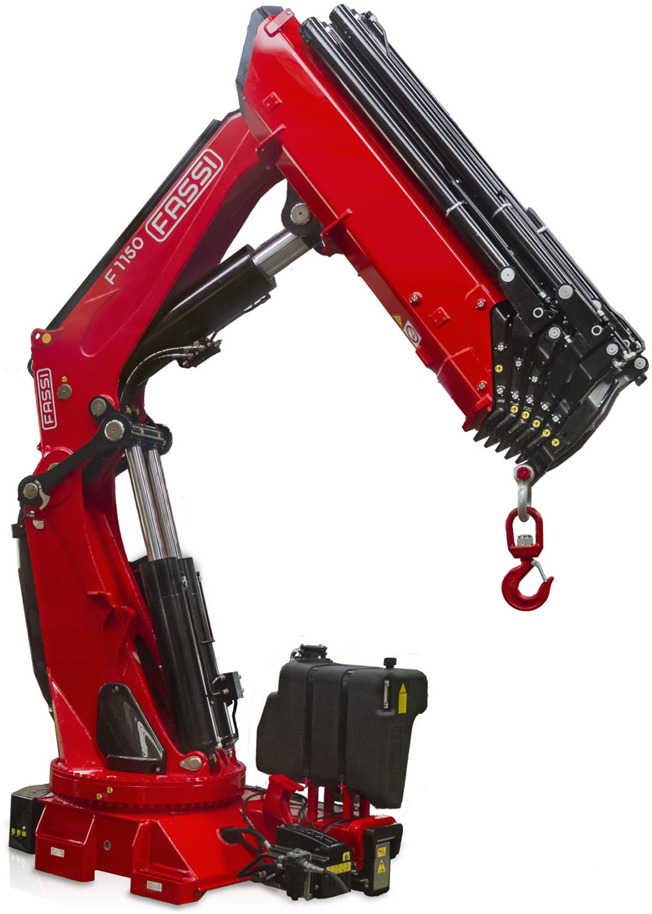
F1150 model: 4 Dof reachability: 19.5 [m] Max. torque: 1,000 [kNm].

Figure 6 shows the pose tracking results for the F1150 model, which is shown in Figure 7. From the top to the bottom are the speed input reference, the comparison between the target pose resulting from the speed integration and the actual pose of the crane, and the tracking error. The RMS for the tracking error is equal to 0.02[m] for all the Cartesian directions. The peaks of tracking errors are approximately 0.1[m] and appear at the change of speed direction.

The tracking errors with respect to the cranes’ maximum elongation are less than 1% for both the M20 and F1150 models. Those errors are comparable with other studies on crane boom control for articulated cranes, such as La Hera et al. (2023) and Lee and Brell-Cokcan (2020). We evaluate those results as acceptable for the crane operability.

### 2.3 Human–robot interfaces

In Section 2, we highlighted the need for a transparent interface to provide the user with a first-person experience while remotely controlling the crane in an intuitive and immersive way. This implies a tight integration with the control module and an accurate design of the crane exteroceptive perception system.

**FIGURE 8:**
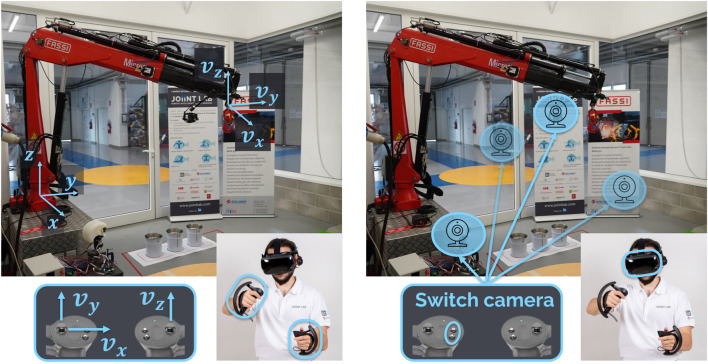
Example of HRI for crane remote operation: on the left is the command interface, and on the right is the perception interface.

Crane’s control inputs**:** the introduction of crane’s boom tip control allows defining a standard for the regulation of the crane’s movement since the Cartesian speed (control input) is independent of the crane model. Concerning the physical control interface, the three Cartesian components that describe the boom tip speed are mapped over two analog joysticks. Figure 8 shows the reference frames’ definition and their mapping: the speed components along the *x*–*y* direction are set through the left analog joystick, along the *z* direction on the right one. This approach has been tested on different user interfaces, on handheld VR controllers (as shown in the figure), and on industrial radio controllers with analog controllers.

Preliminary tests have been conducted involving an experienced ( > 10 years) and a naive operator. Results are reported in Table 1. It is worth noticing that the naive user achieved performances similar to the experienced one (in time and trajectory), owing to the introduction of the intuitive control, which is composed of the boom tip control module and joystick interface.

**TABLE 1:** Preliminary crane boom tip control evaluations comparing the performances of an experienced vs. an unexperienced crane operator using the standard control and the newly developed one.

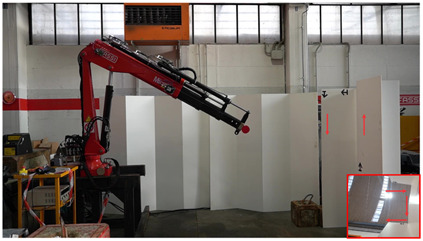 Crane test setup	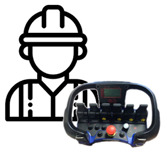 Expert crane operator with standard control	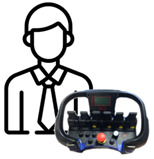 Unexperienced crane operator with standard control	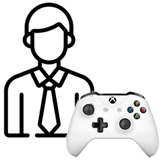 Unexperienced crane operator with crane boom control
Task training time [min]	0	2	0
Attempts	0	3	0
Execution time [min]	2	2	2
Abs of max error [m]	0.05	0.16	0.04

Crane’s perception: the visual feedback of the crane’s surrounding environment is provided by a VR headset or head-mounted display (HMD). This is a highly immersive tool that completely disconnects users from their immediate surroundings, allowing them to focus only on the crane. On the crane side, we envision multiple cameras to provide the different points of view that are useful for task completion. A camera mounted on the crane base (e.g. truck) gives awareness of the global environment, such as obstacles or people, while a camera on the boom tip provides a closer focus on the target. They can be mounted on a fixed or a mobile actuated support. When the camera’s support is actuated, we can tilt the camera to track the orientation of the operator’s head as an extension of the operator’s neck. This choice allows a wider visual exploration of the environment in a natural and immersive way. This perception aids in providing a remote crane operator with full awareness of the crane and surrounding environment. Figure 8 shows an example of the cameras’ setup, with the HMD returning the visual to the operator and the possibility to switch between different cameras through the controllers’ input.

## 3 Experimental validation

The proposed remote Cranebot was tested during the 33rd edition of Bauma, the world’s leading trade fair for construction machinery. This context gave us the opportunity to relate with professionals in the field and crane operators and collect feedback from them directly testing the solution.

### 3.1 Experimental setup

**FIGURE 9:**
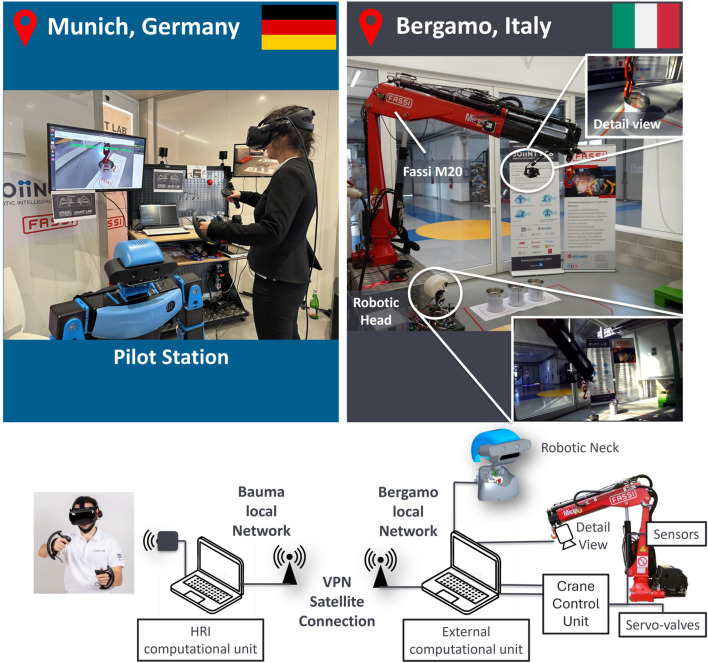
Experimental setup architecture: physical implementation (at the top) and related functional scheme (at the bottom). On the top left is shown the crane operator and HRI in Munich. On the top right is the crane M20 model in Bergamo. In the detail views are shown the perspective of a mobile camera mounted on a robotic actuated head and of a fixed camera mounted on the crane’s boom tip.

The test consisted of a remote crane operation over 350 km between Bergamo and Munich (Figure 9). The operator interface included a Valve Index VR set with a pair of controllers. Using the analog joysticks, the operator could remotely move the crane using the Cartesian control described in Section 2.1, while the HMD would return the visual feedback of the crane environment.

On the robot side, we used the Fassi crane model M20 (specifications reported in Section 2.2.2), which equipped two cameras showing different points of view. The first one was located near the crane and mounted on an actuated neck, whose design is based on that of Negrello et al. (2019). The movement of the robotic neck matched the operator’s head motion, providing a first-person experience. Through this camera, the user has a broad perspective of the crane and an overview of the surrounding environment, including obstacles, item positions, and target release location. Another camera was rigidly fixed on the crane boom to have a detailed view of the item to be picked up/placed. In any moment, using the controllers, the user is able to change the camera view to see either the object in detail or the obstacle around the crane. Additionally, the user was able to monitor a set of real-time safety-related information projected on the HMD, such as the activation and connection to the crane’s movements.

The bottom part of Figure 9 shows the setup’s network: on the crane side, an external computational unit is wired connected with the crane’s Crane Control Unit, the robotic neck, and the cameras, and runs the Cranebot control and the control of the robotic neck. It streams the cameras’ image and the crane state. On the operator side, another computational unit elaborates and transmits the physical inputs of the operator, receives the cameras’ stream, and projects it onto the HMD.

The connection between the two computational units was established over the internet. As mentioned in Section 2, the choice of the network infrastructure is particularly critical for the video stream, which represents the greatest amount of exchanged information. The minimal requirements for our system are 5Mb bandwidth and latency under 200[ms] . Since these performance parameters, although compatible with common internet connectivity standards, were not guaranteed by the fair network, during the tests, we relied on Starlink satellite technology. Clearly, this solution is not the only one that allows an effective connection; still, this experience proves that the technology to enable remote operation is already commercially available on the market, even in conditions where standard connectivity is limited. In addition, to manage the possibility of network degradation, we implemented a safety feature that, in case of communication losses, deactivates the remote control and holds the current state of the crane and displays a warning message to the operator interface.

### 3.2 Test description

**FIGURE 10:**
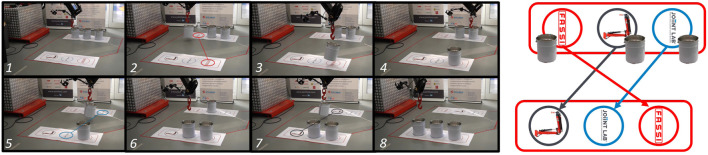
Photo-sequence showing the different phases of the task proposed to the volunteer testers on the left and the top view of the symbols to be matched on the right.

For the test, we simulated a repeated pick-and-place action, a typical crane task in construction sites, such as downloading a truck. Figure 10 shows a sequence from one of the test sessions: three buckets are aligned on one side of the room, and the user has to move them with the crane to the opposite side, matching the symbols printed under them. We asked to complete the task in the shortest possible time, having a hard constraint of 4 min, provided by a timer projected on the operator’s screen, and beyond this limit, the game ended. To be successfully remotely performed, this task required enhanced perception capabilities and fine control of crane’s movements, given the size of the hook and the bucket’s handle. An extract of the experiments is reported in the video provided as Supplementary Material.

All the subjects received a brief guided training to gain confidence on the system. The first part of the training focused on the Cartesian speed commands; most users, especially the experts, were not familiar with the concept of moving the boom tip directly, and in this case, the image was projected on a flat screen in from of them. Then, they were trained with the full setup with HMD, trying the functionality of switching between the camera’s perspective and exploring the room with the neck. This first phase lasted approximately 3 min. Finally, they were asked to complete the task once within the given time.

### 3.3 Usability evaluation

To assess system’s usability, we asked the users to complete a subjective questionnaire known as the NASA Task Load Index (NASA-TLX), which is a widely recognized survey-based measure of workload (Grier, 2015).

**FIGURE 11:**
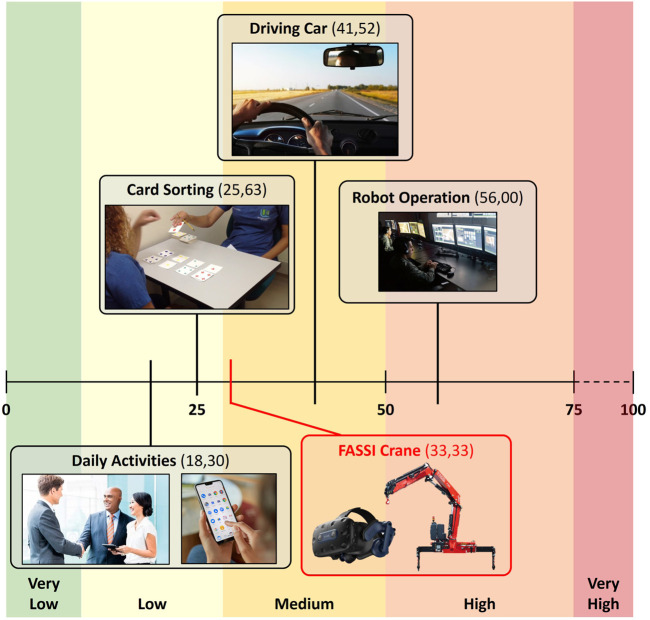
NASA-TLX score comparison between the remote crane control system and other activities of various complexities (Grier, 2015).

The global score of the NASA-TLX comprises six subscales, namely, mental demand, physical demand, temporal demand, frustration, effort, and performance, which are weighted to derive an overall evaluation. To assess each dimension, participants typically rate them on a 0–100 scale, where 0 represents the minimal workload or demand, and 100 represents the maximum ones. For example, engaging in conversation, completing a telephone inquiry, and using a home medical device as part of daily activities may result in a NASA-TLX score of approximately 18.30 (Grier, 2015) (Figure 11). Test scores above 50 are considered high, indicating a significant mental workload.

## 4 Discussion

In total, 78 professionals, ranging between 20 and 60 years old with different levels of experience, tested the remote Cranebot system. Among all the volunteers, approximately half were experienced crane operators (+10 years of experience), while approximately 15%–20% had little experience or did not operate cranes as the main duty in his/her job. Only few had previous experiences with virtual reality. Out of the 78 questionnaires, three were not included in the evaluation since they were not complete.

The general feedback received from users was positive; after a brief training phase, all users, independent of the background and experience, were successful in moving at least one bucket. In general, we noticed that the more experienced users were faster in accomplishing the task, moving more than one bucket in the given time (4 min), and the best user was able to complete the full task in only 2 min.

Concerning user experience, during the tests, several people highlighted the difference between the Cranebot control interfaces and the standard ones in terms of range of motion (ROM). Given that the analogic levers map a velocity reference from 0% to 100%, the standard control station provides more sensitivity to the user, having a more extended range of motion.

Concerning the VR headset, although recognizing the benefit of immersive view and the possibility of reorienting the third-person camera, few users experienced some sickness and expressed their preference for a standard monitor with a fixed point of view.

All the users positively evaluated the possibility to switch between different perspectives, passing alternatively from the full overview to the detailed view of the crane tip.

Among other aspects, we observed that previous experience with gaming, virtual reality, or drones that adopt similar interfaces and control paradigm had an impact on the user experience. The fact that these technologies are becoming more and more popular should be taken into consideration by machine constructors since future users may be more familiar with such interfaces.

From a quantitative perspective, the global score obtained is 33.33 of the NASA-TLX index, which, compared to standard daily activities, is fairly low. The overall assessment falls within the ‘medium’ range on the NASA scale, and as an example, we report in Figure 11, the NASA value of other activities such as standard robot operation (score 56) and driving a car (score 41) (Grier, 2015).

**FIGURE 12:**
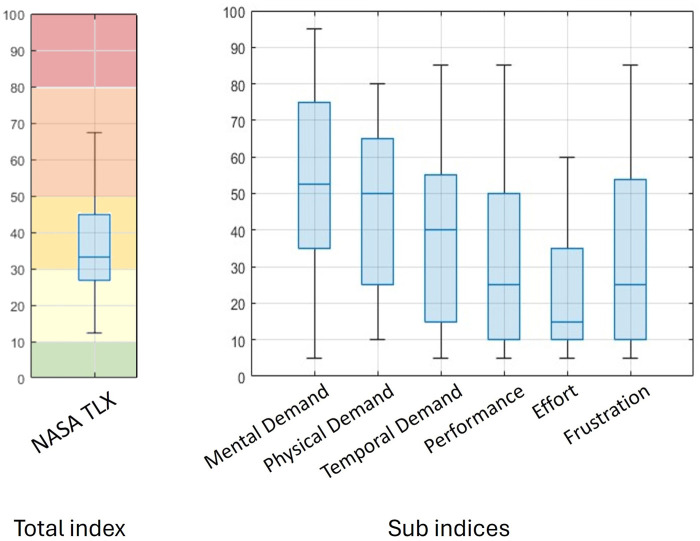
NASA-TLX rating of remote Cranebots resulting from experimental tests at Bauma. On the left is the overall score, and on the right are the rating values of the six NASA sub-scores.

Figure 12 reports the detailed results of the NASA-TLX test. In particular, on analyzing the sub-scores, it turns out that the most demanding aspects of the system are the required *mental demand* and *effort*.

The first parameter refers to the level of mental and perceptual activity required during the task, such as thinking, deciding, calculating, and remembering. In the proposed task, this relates to the capacity to recall the functionality of each control button and proficiently manage the crane. On the other hand, the *effort* dimension provides a comprehensive measure of the overall challenge in completing the task, involving both mental and physical aspects.

Low scores, conversely, were obtained for both the *physical demand* and *frustration* sub-indices, indicating that the tasks require low physical effort and do not induce feelings of stress or annoyance.

Finally, the performance score indicates how successful/satisfied the operator was with his/her performance in accomplishing the goals. In these tests, the average of the performance score is approximately 25, which indicates a low satisfaction in the result obtained.

This value is in contrast with the feedback collected when speaking with the users during the tests. Given the high precision required, most of the participants were skeptical about the task feasibility before trying, while after the task, they were surprised by the system’s simplicity and reported a good level of satisfaction overall. A possible interpretation of this result could be related to the fact that many users reported a personal bias due to a long lasting experience with standard controls and blamed themselves rather than the system for not performing as expected. We also observed that each parameter exhibits a high dispersion of results. This variance may be attributed to the diverse backgrounds of the users, ranging from individuals with no experience in managing a crane to others who are experts in the field, as well as the significant age differences among the participants.

## 5 Conclusion

Our goal was primarily to improve construction operators’ work conditions and safety, providing them with more intuitive control interfaces. To this aim, we proposed Cranebots, a novel system for intuitive remote crane control. Our solution is composed by a scalable boom tip control module, tested on different Fassi crane models (M20 and F1150), with tracking errors lower than 1% of the total cranes’ elongation, and immersive HRI systems to enable first-person experience while remotely controlling the crane. To the best of the authors’ knowledge, this is the first experimental solution combining immersive interfaces with remote crane boom tip control.

The proposed system was tested at the 33rd edition of Bauma, the world’s leading trade fair for construction machinery, where 78 professionals were involved in the system usability test, and it was possible to collect users’ feedbacks and quantitative measurements. Cranebots scored overall 33.33 points on the NASA-TLX test, which assesses a low-medium workload.

Beyond this, we believe that the proposed system could be easily ported on other construction machines, impacting different aspects. On the machine side, it can improve their overall efficacy and efficiency, and from a business perspective, it allows exploring new market opportunities.

Future research work should include the exploration of advanced interfaces with richer sensory feedback to enhance operators’ situational awareness and the development of algorithms for shared autonomy to provide further support to crane users.

## Data Availability

The raw data supporting the conclusions of this article will be made available by the authors, without undue reservation.

## Footnotes

1
https://www.joiintlab.com/
